# Plant-Derived Functional Ingredients in Pet Nutrition: Phytochemical Classification, Mechanisms, Efficacy, and Application in Dogs and Cats

**DOI:** 10.3390/ani16071034

**Published:** 2026-03-27

**Authors:** Atcharawan Srisa, Pitiya Kamonpatana, Khwanchat Promhuad, Phanwipa Wongphan, Anusorn Seubsai, Phatthranit Klinmalai, Nathdanai Harnkarnsujarit

**Affiliations:** 1 Department of Packaging and Materials Technology, Faculty of Agro-Industry, Kasetsart University, Bangkok 10900, Thailand; atcharawan.sri@ku.th (A.S.); khwanchatpromhuad@gmail.com (K.P.); phanwipa.w@ku.th (P.W.); 2 Department of Food Science and Technology, Faculty of Agro-Industry, Kasetsart University, Bangkok 10900, Thailand; fagipyk@ku.ac.th; 3 Department of Chemical Engineering, Faculty of Engineering, Kasetsart University, Bangkok 10900, Thailand; fengasn@ku.ac.th; 4 Faculty of Agro-Industry, Chiang Mai University, Samut Sakhon 74000, Thailand; 5 Center for Advanced Studies for Agriculture and Food (CASAF), Kasetsart University Institute for Advanced Studies (KUIAS), Kasetsart University, 50 Ngam Wong Wan Rd., Latyao, Chatuchak, Bangkok 10900, Thailand

**Keywords:** polyphenols, plant extracts, pet food, functional, pet health

## Abstract

This review classifies plant-derived functional ingredients in pet food according to phytochemical groups and application forms, including direct oral supplementation and incorporation into complete diets. Polyphenols and plant extracts exert prominent antioxidant (singular), anti-inflammatory, immunomodulatory, and microbiome-regulating effects. Microalgae and omega-3 sources support lipid metabolism, cardiovascular function, and skin integrity. Cannabinoids demonstrate dose-dependent responses in dogs, while cats generally tolerate long-term administration and exhibit notable benefits in chronic pain management. Combinations of botanical extracts with complementary bioactives and fermented botanical preparations exhibit multi-target functionality, with dogs showing pronounced biochemical and microbiome modulation, whereas cats display more behavioral and functional improvements. Phytochemicals operate through integrated multi-level regulation, including activation of antioxidant enzymes, modulation of inflammatory cytokines and T-lymphocyte ratios, microbial metabolic shifts toward short-chain fatty acid production, and regulation of lipid metabolism. Dogs demonstrate marked effects on hepatic function, reproductive resilience, microbiome diversity, CD4+/CD8+ balance, and cholesterol control. In contrast, cats show greater benefits in inflammation reduction, pain relief, intestinal integrity, and long-term safety. These species-specific responses underscore the importance of precision formulation and highlight the emergence of plant-based “pharma-pet nutrition” integrating nutritional and biochemical strategies for targeted health promotion.

## Simple Summary

Modern pet nutrition is evolving toward the use of phytochemical-rich, plant-derived ingredients that provide health benefits rather than basic nutrition. This review summarizes and categorizes plant-derived bioactives used in dog and cat food, explaining their mechanisms of action, usage forms, and outcomes from feeding trials. Evidence from these studies indicates that many plant-derived substances can enhance antioxidant activity, balance gut microbiota, support metabolic function, and reduce inflammation. However, dogs and cats have different physiological and metabolic characteristics, which leads to different responses to plant-derived ingredients. Understanding these differences is essential for the development of safe and effective pet food formulations, which can support preventive health care and enhance the long-term quality of life for pets.

## 1. Introduction

The use of plant-derived compounds as dietary supplements is becoming increasingly popular in companion animal nutrition, particularly in dogs, where phytogenic ingredients are increasingly incorporated as biofunctional components beyond basic nutrient provision [1,2,3]. Growing consumer awareness of personalized pet care has accelerated the development of functional pet foods targeting gastrointestinal health, dermatological conditions, metabolic disorders, cognitive decline, and immune balance. Consuming plant-derived phytochemicals as botanical supplements may support metabolic balance in animals. In this review, “metabolic balance” refers to metabolic homeostasis, reflected by stable regulation of glucose and lipid metabolism, energy utilization, and related biomarkers (e.g., blood glucose, lipids, liver-associated markers, and body composition measures) [4,5]. Functional formulations commonly include prebiotics, polyphenols, and herbal extracts, and their long-term pet health care success depends on predictable efficacy, safety, and biologically relevant dosing strategies [1,4,6,7]. However, species-specific physiology necessitates careful evaluation. Cats are obligate carnivores requiring animal-derived nutrients for survival, whereas dogs have adapted to omnivorous diets that include starch-rich plant ingredients [8]. These metabolic differences influence the utilization, safety, and functional outcomes of plant-derived bioactives. For example, beta-carotene is not essential for dogs but can affect their immune response, while taurine plays a critical role in reducing lipid accumulation in the livers of cats [9]. These differences indicate significant gaps and considerations for plant-based supplements and beneficial ingredients, particularly the use of phytochemicals between omnivorous dogs and carnivorous cats [10].

Polyphenols are bioactive compounds derived from plants that have proven antioxidant properties and play a role in preventing aging in animals, as evidenced by reductions in lymphocyte and globulin levels. The properties of plant-based dietary supplements include antioxidant, anti-inflammatory, immune-modulating, and gut microbiota-balancing effects, which contribute to the prevention or treatment of various diseases and behavioral disorders in pets [6,11,12]. In companion dogs and cats, polyphenols have shown positive effects on gastrointestinal function and microbial composition, as well as on obesity, glucose control, fat metabolism, and cardiovascular health. New evidence also supports their role in slowing cognitive decline and modulating immunity, highlighting their value as beneficial ingredients in pet nutrition [5,13]. The safety of metabolism of natural plant extracts by dogs and cats depends on several factors, including the type of extract, dosage, extraction method, health status, and individual variability of the animals. These considerations are particularly important when considering the physiological and metabolic differences between dogs and cats. The appropriate dosage must be adjusted according to the specific extract, the pet’s weight, and the proportion in the daily diet [14,15,16,17].

Recent reviews have summarized plant extracts in dogs and cats with emphasis on metabolism/toxicology and general health effects [17] or have focused on phytogenic polyphenols with an emphasis on canine health outcomes [4]. These studies provide valuable insights into functional properties and potential health benefits. However, there is a lack of a combined overview across phytochemical classes, application or mode of incorporation, and species-specific responses for the illustration of real pet food formulations. There remains a limited synthesis of how bioactive compounds are applied in pet nutrition, including their mode of incorporation (dietary inclusion vs. oral supplementation), effective dosing ranges, and outcomes from controlled feeding trials in both dogs and cats. Furthermore, comparative evaluation between species, especially considering the distinct metabolic differences between omnivorous dogs and obligate carnivorous cats, is still insufficiently addressed.

This review focuses on plant-derived functional ingredients with an emphasis on phytochemical-based ingredients used in companion animal nutrition, including phytochemical classification, dosing, application approaches (dietary incorporation or oral administration), and outcomes from controlled feeding trials in dogs and cats. The functional mechanisms and reported effects on immune function, metabolic stability, gastrointestinal health, neurological function, and antioxidant-related outcomes after short- or long-term feeding are summarized. This review demonstrates how plant-based functional additives may support preventive health strategies and contribute to evidence-based nutritional management in companion animals. By emphasizing evidence from feeding trials, practical application formats, and species-specific responses, this work aims to advance current knowledge toward more precise and evidence-based formulation of functional pet foods.

Literature search and study selection: A focused search was conducted in Scopus using keywords related to dogs/cats, pet foods, feeding trials, and plant-derived functional ingredients (dietary inclusion or oral administration). Studies were included if they evaluated plant-derived ingredients or plant-derived phytochemicals in dogs and/or cats and reported functional outcomes (clinical scores, biochemical markers and/or microbiota/fermentation measures, etc.), with dose and duration recorded when available. Included studies were then grouped by dominant bioactive class or ingredient type and by application format to support evidence-based formulation considerations.

## 2. Classification of Plant-Derived Functional Ingredients in Pet Food

Interest in functional pet foods has increased, and current formulations aim to include bioactive compounds that deliver measurable physiological benefits while maintaining safety and stability throughout processing and consumption. Plant-derived ingredients are a key source of functional compounds because they provide a wide range of phytochemicals that are not essential nutrients but still influence biological processes. In companion animals, these phytochemicals have been associated with antioxidant activity, modulation of immune responses, and reduction of inflammation [4,18]. In this review, classification is based on how plant-derived functional ingredients are used in pet foods and the type of bioactive effect supported by dog/cat feeding trials. Most of the sections focus on phytochemical-rich ingredients (polyphenols, botanical extracts, cannabinoids, and microalgae).

### 2.1. Polyphenols

Polyphenols are one of the most diverse groups of plant-derived phytochemicals and are widely studied in companion animal nutrition. Their functional roles include antioxidant activity, modulation of gut microbiota, and anti-inflammatory effects. As shown in Table 1, studies use two main application formats: orally administered supplements or ingredients incorporated into complete diets. Results are generally positive for biomarker outcomes (inflammation, oxidative stress, and microbiota), but the evidence strength varies due to different models (healthy vs. disease vs. stress), different dose reporting, and small sample sizes in several trials.

For orally administered supplements, resveratrol is a plant-derived polyphenol naturally present in grapes, berries, and peanuts [19]. In greyhound dogs, 7-day oral resveratrol (1000 mg/day) improved blood pressure stability during hemorrhagic stress without increasing bleeding tendency. But it did not reduce kidney injury biomarkers or kidney damage [20]. Grape seed proanthocyanidin (30 mg/kg BW/day) mitigated intestinal inflammation in Labrador Retrievers with mild inflammatory bowel disease (IBD). It improved inflammatory profiles and decreased intestinal permeability, and fecal microbiota transplantation results supported a microbiome-related mechanism [21]. Polyphenols from a grape and blueberry extract given for 24 weeks (4, 20 or 40 mg/kg BW/day) were well tolerated in long-term use and showed no biochemical or clinical signs of hepatic or renal toxicity in Beagle dogs. The extract contained multiple polyphenol classes, and the metabolites were detectable in plasma, providing stronger evidence for their long-term safety in canine nutrition [22]. Similarly, mandarin orange peel flavonoids showed potential to improve gut microbiota and cognitive behavior in Miniature Dachshunds, but the evidence is still preliminary due to the very small sample size [23].

Incorporation of polyphenols into complete diets has also been investigated. Green tea polyphenols mixed directly into high-fat canine diets (0.48%, 0.96%, or 1.92% for 18 weeks) reduced weight gain and inflammatory cytokine expression and was associated with gut microbiota composition change [13]. Silybin, the key flavonolignan in milk thistle extract, is incorporated into commercial canine diets as a hepatoprotective ingredient [24]. Purified silybin or commercial hepatoprotectant at 12.75 mg/10 kg BW did not alter nutrient digestibility or clinical parameters in healthy dogs. In beagles with hepatopathies, the same dosage lowered serum liver enzyme activities and reduced liver-associated microRNAs, including miR-122, indicating improved hepatic status, which in turn indicated improved liver function [24]. Gallnut tannic acid (2.5 g/kg basal canine diets) also demonstrated multiple benefits, including reduced stress-related diarrhea, oxidative stress and inflammatory responses in Beagle dogs. However, these findings are based on short-term studies under environmental stress for 14 days with a limited sample size [5]. Dietary anthocyanins from honeyberry, providing 10.50 mg cyanidin-3-O-glucoside/100 g extruded food, improved cognitive dysfunction scores and reduced serum amyloid beta oligomers in elderly dogs after feeding for 90 days. But the evidence remains limited due to the small sample size and variability among animals [25].

Polyphenols show promising functional roles in companion animals with generally consistent trends for gut microbiota and inflammation-related biomarkers. However, the strength of evidence varies across compounds. Many studies are limited by small sample sizes, short durations, and specific models or conditions (e.g., mild IBD, stress exposure, and cognitive dysfunction). Differences in study design, ingredient form (purified vs. extract), dose metrics, and endpoints reduce direct comparability across papers. Therefore, more standardized and longer-term feeding trials with clearly reported doses and clinically relevant outcomes are needed to confirm practical applications and strengthen evidence-based formulation guidance.

**Table 1:** Polyphenol-rich ingredients evaluated in dog and cat feeding trials: delivery format, dosing, targeted function, and key reported outcomes.

Functional Ingredient	Mode of Incorporation/Usage	Supplementary Form	Functional Ingredient Dose	Experimental Diet	Targeted Function	Reference
Resveratrol (micronized trans-resveratrol powder)	Administered orally	Dietary supplementation (not specific form)	Oral resveratrol 1000 mg per day for 7 days Estimated intake > 10 mg/kg/day	Dogs were housed individually and fed the same intestinal diet twice daily, receiving either no supplementation (control, *n =* 6) or oral resveratrol (resveratrol group, *n* = 6).	Improved hemodynamic tolerance to hemorrhage	[20]
Grape seed proanthocyanidin (GSP)	Administered orally	Dietary supplementation (dissolved in 10 mL normal saline on an empty stomach)	30 mg/kg body weight GSP daily	36 adult Labrador Retrievers (12 healthy, 24 mild IBD) were housed individually and fed an identical basal diet twice daily. Healthy dogs served as the control group, while IBD dogs received the basal diet with or without grape seed proanthocyanidin.	Reduction of intestinal inflammation Improved intestinal barrier function Gut microbiota modulation Bile acid metabolism modulation	[21]
Mixed grape and blueberry extract	Administered orally	Extract administered orally in gelatin capsules	Tested dosages: 4, 20, and 40 mg per kg body weight per day	Four groups of 6 adult dogs (5 males and 1 female per group) were housed individually and fed the same basal diet twice daily. The control group received no supplementation, while the treatment groups received gelatin capsules containing polyphenol-rich grape and blueberry extract at 4 mg/kg, 20 mg/kg, or 40 mg/kg body weight per day mixed with their daily meal.	Support renal safety Support hepatic safety Systemic exposure confirmed (plasma metabolites detected)	[22]
Mandarin orange peel extract containing flavonoids (particularly hesperidin and nobiletin)	Administered orally	Dietary supplementation (used in gelatin-based cube formulation for dosing)	1 g of standardized citrus peel extract administered twice daily (2 g/day total)	Four healthy adult Beagle dogs (*n* = 4) were fed their regular commercial diet and received a single 4 g oral dose of the mandarin peel extract, followed by a washout. The same dogs then received 1 g of the extract twice daily for 14 days, using each dog’s baseline values as its own control.	Gut microbiota modulation Flavonoid absorption/exposure (plasma detection)	[23]
Green tea polyphenols (GTP) extracts including catechin (C), EGC, gallic acid, tetrahydrofuran (THF), EGCG, (−)-gallocatechin gallate, ECG, and tea caffeine	Mixed directly into diet at specified inclusion levels	Dog food (normal diet or high-fat diet formulation)	Low dose: 0.48 g/kg diet Medium dose: 0.96 g/kg diet High dose: 1.92 g/kg diet	Dogs were assigned to five groups (*n* = 6 per group): Normal diet (control). High-fat diet (≥45 g/kg added fat). High-fat diet supplemented with green tea polyphenols at 0.48% (low dose), 0.96% (medium dose), or 1.92% (high dose).	Reduced weight gain or anti-obesity function Gut microbiota modulation Reduction of intestinal inflammation	[13]
Flavonolignan (silybin; active component of silymarin)	Commercial hepatoprotectant containing silybin (HEP) or pure silybin (SIL) added as a supplement to the basal dry diet	Commercial basic diet (lamb-based formula)	12.75 mg silybin per 10 kg body weight (both pure silybin and commercial hepatoprotectant formulations)	Dogs were housed individually and fed the same commercial basal diet twice daily. The control dogs received the unsupplemented diet, while the HEP and SIL groups received the same diet supplemented with either a commercial silybin preparation or pure silybin at 12.75 mg per 10 kg body weight.	Improved liver function markers Hepatic stress modulation (ALT/AST and miR-122 reduction)	[24]
Hydrolysable tannin (tannic acid powder from gallnut)	Tannic acid powder thoroughly mixed into the basal diet before feeding; fed individually to each dog	Dry extruded dog food	Used dosage: 2.5 g/kg of basal diet Equivalent intake: 0.5 g tannic acid per 200 g feed per dog per day	Dogs were housed individually and fed the same commercial extruded basal diet twice daily. Thirteen Beagle dogs were enrolled and assigned to a stress control group (*n* = 6) receiving the unsupplemented diet or to a tannic acid group (*n* = 7) receiving the same diet mixed with gallnut tannic acid at 2.5 g per kg of diet for 14 days.	Reduced stress-related diarrhea Stress-response modulation (cortisol reduction) Gut microbiota modulation Increased fecal butyrate and SCFA support	[5]
Hydrolyzed honeyberry (rich in anthocyanin cyanidin-3-O-glucoside)	Powdered anthocyanin extract incorporated into commercially extruded dry pet food	Dry extruded dog food	Used 10.50 mg cyanidin-3-O-glucoside/100 g pet food	Nine elderly dogs of mixed breeds (five neutered males and four neutered females) were housed and fed individually once daily according to body weight for 12 weeks. All dogs received the same commercial diet, and food intake was monitored weekly. The anthocyanin group received the diet mixed with cyanidin-3-glucoside supplement, while the control group received the same diet without supplementation.	Improved cognitive dysfunction score Reduced serum amyloid-beta oligomers	[25]

Note: In the “Targeted Function” column, only outcomes reported as statistically significant versus the appropriate control (*p* < 0.05) are listed.

### 2.2. Plant Extracts

Plant extracts are complex mixtures of bioactive compounds derived from whole plant materials (e.g., leaves, roots, seeds, and fruits). In pet nutrition, they are used either as oral supplements (controlled dosing) or dietary inclusions (routine feeding) to support functions such as metabolic, gastrointestinal, immune, and systemic health beyond basic nutrient provision [2,17]. Table 2 summarizes plant extracts used in dogs and cats, highlighting the administration format, dose range, and reported functional outcomes.

Orally administered supplements have been widely used to evaluate the systemic effects of plant extracts under controlled dosing conditions. Black ginseng enriched in ginsenoside Rg5 tablets (400 or 800 mg/10 kg BW/day for 8 weeks) induced marked alterations in serum amino acid profiles in Beagles [26,27]. This supports systemic metabolic modulation, but these findings are mainly based on metabolomics rather than clinical outcomes. *Melissa officinalis* extracts improved behavioral scores and reduced stress-related responses in Beagles, indicating potential calming effects, but the results are based on short-term trials [28]. *Echinacea angustifolia* extract (1 mL of 5% twice daily for 60 days) increased hematological indices, phagocytic activity, and serum IgM levels, demonstrating systemic immune stimulation in dogs. However, confirmation in larger trials with clinically relevant endpoints is still needed [29]. For reproductive support, *Lasia spinosa* Thwaites extract (10 mg/kg BW/day for 60 days) improved post-thaw sperm motility and viability without altering hematological or serum biochemical parameters, supporting short- to mid-term safety for canine sperm cryopreservation [30]. In a clinical setting, *Elymus repens* extract (2 mL of 0.48 mg/kg every 12 h for 21 days) reduced urinary density and crystalluria while maintaining stable blood parameters in dogs diagnosed with urolithiasis [31]. Compared with dogs, evidence in cats is still limited but highlights metabolic applications. Unripe avocado gel altered gut microbiota composition and fecal metabolomic profiles in naturally overweight/obese cats [12], while a quercetin-rich plant extract mixture reduced inflammatory markers and altered lipid-related biomarkers in obese cats [32]. However, these feline studies were small and short-term and do not provide standardized dosing guidance for broad clinical use.

Dietary inclusion studies better reflect real feeding practices, but results vary depending on formulation, duration, and health status. Curcumin-coated kibble (6 mg/dog/day; 1.5 mg/kg BW) improved antioxidant-related responses and showed anti-inflammatory activity in Beagles [33], although interpretation is complicated by health disturbances during the trial due to reduced lymphocyte counts and lowered serum globulin concentrations. Whole-ground-flaxseed- or sunflower-seed-coated kibble at 3% induced transient improvements in skin and hair coat condition and increased serum 18-carbon polyunsaturated fatty acids, including α-linolenic acid and linoleic acid, indicating short-term modulation of skin lipid status [34]. A semi-moist diet containing plant extracts (echinacoside, anthocyanidin, curcumin, and silybin) modulated metabolic and immune biomarkers without adverse effects in a 60-day trial [35]. But the groups were not directly comparable because differences in the baseline conditions (e.g., arthrosis for curcumin and hepatopathy for silybin) and endpoints across the groups limited direct comparison. In dogs with atopic dermatitis, a functional canine diet with *Siraitia grosvenorii* residual extract (6.5 g/kg) improved skin barrier function and reduced pruritus and lesion severity without adverse effects [36]. However, because the study used a self-controlled before–after design (no parallel control group), randomized parallel-diet trials are needed to confirm that the improvements were attributable to the diet. In Rottweilers, rosemary and basil leaves reduced serum glucose by up to 31% and inhibited amylase activity [37]. In British Shorthair cats, rosemary extract (0.1%; <100 Da fraction) reduced ammonia and hydrogen sulfide emissions during a 30-day feeding trial, supporting a deodorizing application [38]. However, evidence is currently limited to one breed and a controlled feeding setup, so generalization to other breeds or clinical pets needs confirmation. A multi-herb supplement (Bioticks^®^, 9 mL/kg) reduced flea counts without adverse effects over 5 months and with good tolerance [18]. The evidence is relatively strong because the trial was randomized, double-blind, and placebo-controlled, although household conditions can introduce variability and the active components cannot be isolated. In vitro fermentation demonstrated modulation of microbial metabolic activity and reduced volatile compound production in canine and feline fecal cultures [39], but feeding trials are needed to confirm real-animal outcomes. Finally, a crossover feeding study showed that a natural antioxidant blend (essential oils + vitamin E; 1% of feed) maintained feed stability and reduced reactive oxygen species (ROS) in Beagles over 28 days without metabolic or hematological alterations [40]. But this evidence remains short-term and breed-limited.

In summary, plant extracts are applied in pet nutrition either as controlled oral supplements or as diet inclusions, with reported outcomes ranging from metabolic and immune biomarkers to practical functions such as skin support, odor reduction, and flea control. However, results are not equally strong across studies because designs, extract compositions, and endpoints vary, and evidence in cats remains limited, so broader and better-controlled feeding trials are still needed.

**Table 2:** Botanical extracts evaluated in dog and cat feeding trials: delivery format, dosing, targeted function, and key reported outcomes.

Functional Ingredient	Mode of Incorporation/Usage	Supplementary Form	Functional Ingredient Dose	Experimental Diet	Targeted Function	Reference
Plant extracts						
Black ginseng extract (*Panax ginseng*)	Administered orally	Tablets	Medium dose (BG-M): 400 mg per 10 kg body weight per day High dose (BG-H): 800 mg per 10 kg body weight per day	Dogs were housed individually and fed the same commercial laboratory feed once daily. Twelve healthy Beagle dogs were allocated into three groups of four: Control group receiving the regular basal diet (*n* = 4). Black ginseng medium-dose group receiving 400 mg per 10 kg body weight (*n* = 4). Black ginseng high-dose group receiving 800 mg per 10 kg body weight (*n* = 4).	Dose-dependent serum metabolite shifts (amino acid profile) Candidate anti-inflammatory metabolite signature (glycine, β-alanine)	[26]
Black ginseng extract (*Panax ginseng*)	Administered orally	Tablets	Used dosage: 200 mg black ginseng per tablet; total daily intake per dog adjusted to body weight Final intake: two tablets of black ginseng (400 mg per 10 kg body weight per day)	Four healthy adult Beagle dogs (*n* = 4; two males and two females) were fed individually in separate cages and received a standard laboratory diet at 250 g per day. All dogs consumed the same basal diet and were supplemented with black ginseng tablets at 400 mg per 10 kg body weight per day for 8 weeks. Baseline blood samples were collected before supplementation, with follow-up sampling at weeks 4 and 8.	Serum metabolite profile shift (before and after supplementation) Energy/immune-related pathway signature (metabolomics-based)	[27]
Hydro-alcoholic *Melissa officinalis* extract	Administered orally	Supplemented in powder form	*Melissa officinalis* extract at 200 mg/kg of feed	Twenty healthy Beagle dogs were housed individually and fed the same commercial basal diet. Dogs were randomly assigned to four groups (*n* = 5 per group): a placebo group receiving maltodextrin at 200 mg/kg, a group receiving *Melissa officinalis* extract at 200 mg/kg, a group receiving rosmarinic acid at 10.6 mg/kg, and a group receiving α-casozepine at 225 mg. Supplements were administered with the daily diet.	Improved behavioral stability (handling/behavioral score) Reduced behavioral signs of stress and anxiety	[28]
Hydro-alcoholic *Echinacea* extract	Administered orally	Liquid extracts	1 mL of 5% hydroethanolic extract, twice daily for 2 months	Fourteen mixed-breed male dogs were randomly allocated into two groups: - Echinacea group (*n* = 7) receiving 1 mL of 5% extract twice daily. - Placebo group (*n* = 7) receiving water. Dogs were housed under identical conditions and fed a uniform diet throughout the study.	Immune stimulation (phagocytosis, IgM) Increased hematological indicators	[29]
*Lasia spinosa Thwaites*	Administered orally	*Lasia spinosa Thwaites* powder encapsulated	Individual dose of 10 mg/kg body weight per day in the morning before feeding	Six healthy male dogs received oral *Lasia spinosa* Thwaites supplementation at 10 mg/kg body weight once daily for 7 days (short-term) and for 60 days (long-term). All dogs were fed the same commercial diet and served as their own controls across baseline, treatment, and post-treatment periods.	Improved post-thaw sperm motility Improve sperm cryotolerance	[30]
*Elymus repens* (couch grass) extracts	Administered orally	Commercial solution containing Elymus repens extract	2 mL every 12 h (0.48 mg/kg oral route) for 21 days	Twelve male dogs of various breeds diagnosed with urolithiasis were enrolled and randomly assigned to a treatment group (*n* = 6) receiving *Elymus repens* extract at 0.48 mg/kg orally every 12 h for 21 days or to a control group (*n* = 6) receiving the same excipient formula without extract. All dogs remained in their homes and continued their usual diets with water available ad libitum.	Reduction of urinary crystals Support for urolithiasis management	[31]
D-mannoheptulose-enriched avocado extract	Administered orally	Oral gel dietary supplement	Avocado extract oral gel contained 100 mg/mL Daily dose: 5 mg per kg per day during weeks 1–8, increased to 10 mg per kg per day during weeks 9–16 Placebo gel (maltodextrin + Polox gel) was administered at the same dose schedule	Ten naturally overweight or obese domestic shorthair cats were randomly assigned to receive either avocado extract (*n* = 5) or maltodextrin placebo (*n* = 5) for 16 weeks, while ten lean cats served as unsupplemented controls. All cats were group-housed and fed the same commercial diet, with individual housing only for fecal sample collection.	Gut microbiota modulation (Firmicutes reduction/clustering shift) Fecal metabolite modulation (tryptophan–indole pathway)	[12]
*Rhus verniciflua*, *Ulmus hollandica*, *Polygonatum sibiricum*, *Lycium chinense*, *Ganoderma japonicum*, *Parnax ginseng*	Administered orally	Capsule	60 g of lyophilized Rv-PEM01-99 from 230 g of herb powder Powdered Rv-PEM01-99 (300 mg) was packed in capsule form and supplemented to cats at the dose of 100-120 mg/kg/day (2.5–3.0 mg/kg/day as quercetin) for 4 weeks	Ten healthy mixed-breed cats and four obese client-owned cats were included. Healthy cats were divided into a control group (*n* = 5) receiving the unsupplemented commercial diet or a supplemented group (*n* = 5) receiving Rv-PEM01-99. Obese cats (*n* = 4) continued their regular diets and received Rv-PEM01-99 supplementation for 4 weeks.	Reduced serum amyloid A Improved lipid/liver markers in obese cats (total cholesterol, AST/ALT) Reduced plasma NEFA in healthy cats	[32]
Curcumin extract powder	Added after the extrusion process	Dry extruded dog food	Used dosage: 100 mg per kg of feed Final concentration: 32.9 mg/kg	Dogs were housed individually and fed twice daily with a basal dry extruded diet, receiving either the curcumin-containing diet (*n* = 6) or the control diet without curcumin (*n* = 6).	Enhanced antioxidant enzyme activity/antioxidant capacity Reduced reactive oxygen species Reduced lymphocytes/globulins (anti-inflammatory signal)	[33]
Flaxseed or sunflower seed	Whole ground seed coated on the surface of the kibbled product	Dry dog food (kibble)	Final concentration: 3% on an “as-is” basis	Eighteen mixed-breed adult dogs were enrolled and assigned to either the sunflower oil group (*n* = 9) or the flaxseed oil group (*n* = 9). Dogs were housed individually and fed a basal dry diet according to metabolic energy requirements. All dogs received the same basal diet during a 2-week acclimation, after which each group received the basal diet supplemented with its respective oilseed for 84 days.	Temporary improvement in coat/skin scores Increased serum 18-carbon PUFA profile	[34]
Nutraceuticals extracts: *Vaccinium myrtillus*, *Curcuma longa*, *Echinacea* *angustifolia*, *Sylibum marianum*	Extracts were incorporated directly into the semi-moist diet formulation before feeding	Semi-moist canned dog food	*Vaccinium myrtillus* (anthocyanidins): daily dose of extract 0.2 mg/kg live weight *Curcuma longa* (curcumin): daily dose of extract 6.6 mg/kg live weight *Echinacea angustifolia* (echinacoside): daily dose of extract 0.1 mg/kg live weight *Silybum marianum* (silybin): daily dose of extract 1.5 mg/kg live weight	Dogs were fed the same semi-moist basal diet for 15 days before allocation. A total of 74 dogs were assigned to: Control diet without nutraceuticals (*n* = 21). *Vaccinium myrtillus* group (*n* = 13). *Curcuma longa* group (*n* = 18). *Echinacea angustifolia* group (*n* = 14). *Silybum marianum* group (*n* = 8) All dogs received their assigned diet once daily for 60 days.	Downregulated inflammatory gene targets (*TNF*, *CXCL8*, *NFKB1*, and *PTGS2*) Improved liver-associated markers (decreased ALT, increased paraoxonase) Upregulated *SOD_2_* (antioxidant gene signal)	[35]
*Siraitia grosvenorii* residual extract	Mixed directly into diet formulation before feeding	Functional therapeutic diet for dogs	Functional diet was additionally supplemented with 6.5 g/kg diet of *Siraitia grosvenorii* residual extract	Thirty-two client-owned dogs with chronic atopic dermatitis were enrolled (*n* = 32). Dogs were first fed a standard commercial diet for 12 weeks (control phase), followed by the functional diet containing *Siraitia grosvenorii* extract for another 12 weeks (treatment phase). Each dog served as its own control.	Reduced pruritus score Reduced lesion severity Improved skin barrier (TEWL)	[36]
Rosemary (*Rosmarinus officinalis*) or basil (*Ocimum basilicum*) leaf powder	Herbal powders were added during the coating step of diet manufacture	Extruded basal diet	Rosemary leaf powder at 0.05 percent of diet Basil leaf powder at 0.05 percent of diet Combination diet contained rosemary and basil, each at 0.025 percent of diet	Forty-five Rottweiler dogs (4 months old, 20.5–24.5 kg) were assigned to five experimental groups (*n* = 9 per group; three replicates of three dogs). All dogs were fed the same extruded basal diet, with groups receiving either the unsupplemented diet (control) or the diet fortified with rosemary, basil, or their combination for 8 weeks. Dogs were housed individually with daily exercise access, vaccinated, dewormed, and provided water ad libitum.	Lowered serum glucose (hypoglycemic support) Reduced amylase activity Improved antioxidant indices (increased superoxide dismutase, catalase, and glutathione; decreased malondialdehyde)	[37]
Rosemary extract	Powdered rosemary extract mixed with commercial cat food	Commercial cat food supplemented with 0.1% rosemary extract	0.1% rosemary extract	Nine adult British Shorthair cats were assigned to three groups (*n* = 3 per group). Cats were housed individually and fed commercial cat food twice daily for 4 weeks. The control group received the unsupplemented diet, while the two treatment groups received diets supplemented with 0.1% rosemary extract (RE) and below-100 Da rosemary extract RE1 (RE100).	Reduced ammonia and hydrogen sulfide emissions Reduced urease/uricase activity Increased *Bifidobacterium* and reduced sulfate-reducing bacteria	[38]
Biological plant-based food supplement Bioticks^®^ (thyme, rosemary, lemon balm, fenugreek, wormwood, and lemongrass extracts)	Added directly to the dry diet before packaging	Standard dry diet	Added at 9 mL per kg of diet	Twenty cats were enrolled and divided into two groups: Group A (*n* = 10): Received neutral diet (placebo). Group B (*n* = 10): Received the same diet supplemented with the plant extract formulation. Cats lived under household conditions at three sites; no antiparasitic treatments or environmental modifications were allowed during the study.	Reduced flea counts (long-term control)	[18]
Plant saponins (*Yucca schidigera* extract) and/or hydrolysable tannins (chestnut wood tannins)	Directly incorporated into in vitro fermentation vessels using canine and feline fecal inocula.	The experiment used in vitro fermentation media containing undigested residue from commercial dry extruded dog and cat diets.	*Yucca schidigera* extract (YSE): 0.1 g per liter of culture medium Chestnut tannins (CT): 0.3 g per liter of culture medium Combined treatment: YSE 0.1 g/L + CT 0.3 g/L (doses selected to simulate inclusion of 1 g/kg YSE and 3 g/kg CT in commercial dry extruded pet food)	Dogs: Twelve healthy adult Beagle dogs were housed individually and fed the same commercial basal diet (250 g per day). They were divided into three groups (*n* = 4 each): a control group receiving the regular diet, a medium-dose black ginseng extract group (400 mg per 10 kg per day), and a high-dose group (800 mg per 10 kg per day) for 8 weeks. Cats: Four healthy adult European shorthair female cats (*n* = 4) were fed the same commercial dry diet for 4 weeks before fecal sample collection. Their diet composition was reported, and the undigested residue was used for in vitro fermentation.	Reduced ammonia and putrefactive/volatile metabolites (in vitro) Shifted fermentation metabolite profile (in vitro)	[39]
Essential Oils						
Blend of essential oils + vitamin E including clove essential oil, rosemary essential oil, oregano essential oil and vitamin E (α-tocopherol)	Added to dog food during the fat-coating stage via oil bath application + C27:I27	Dry extruded dog food	Blend composition: clove (6%), rosemary (2%), oregano (1%) and vitamin E (3.3%) and vehicle (soybean oil, 87.7%) Dosage of natural antioxidant was 1% (1000 mg per kg of food produced)	Ten adult beagle dogs were divided into two groups of five. Control group: diet containing synthetic antioxidant (BHT). Natural group: diet containing the essential oil blend at 1%. A crossover design was used: dogs consumed each diet for 28 days with a 15-day washout period on a standard diet. Dogs were fed individually twice daily with water provided ad libitum.	Improvement of feed oxidative stability Reduced circulating ROS Increased glutathione S-transferase (GST) and non-protein thiols Reduced fecal bacterial count (day 28)	[40]

Note: In the “Targeted Function” column, only outcomes reported as statistically significant versus the appropriate control (*p* < 0.05) are listed.

### 2.3. Cannabinoids

Cannabinoids are bioactive compounds derived from hemp and cannabis, including delta-9-tetrahydrocannabinol (THC), cannabidiol (CBD), and cannabidiolic acid (CBDA). In companion animals, these compounds interact with the endocannabinoid system and are increasingly being investigated for roles in pain modulation, behavior, inflammation, and neurological function [41,42,43]. Table 3 summarizes cannabinoid products used in dogs and cats, and most oral administration (oils, chews, capsules, or pastes) emphasizes pharmacokinetics, safety, and species-specific tolerability. A hemp-derived formulation providing 2 mg/kg (50:50 CBD/CBDA) was administered orally to Beagles as 15 mg CBD soft chews and to domestic shorthair cats as CBD-infused fish oil for 84 days. Hematological parameters remained within normal ranges in both species, although cats exhibited lower oral absorption kinetics, indicating the need for species-adjusted dosing strategies [16]. In dogs, a THC:CBD (1:20 ratio) showed that low-to-medium doses were well tolerated and considered an acceptable risk, while clinical sign severity correlated with plasma cannabinoid concentrations [44]. In domestic shorthair cats, an oral THC:CBD (1:20 ratio) was well tolerated at both low and high CBD ratios without adverse effects, but plasma exposure was highly variable and generally lower than in dogs, and dose–plasma concentration kinetics were linear under fasting conditions [45].

As regards functional outcomes in cats (practical relevance), a single 8 mg/kg CBD capsule induced mild, transient sedation and improved handling compliance without altering nociceptive thresholds or physiological parameters in healthy domestic shorthair cats and Persian cats. [46]. However, the study lacked a placebo control and reflected short-term effects only. More recent studies in cats have expanded evaluation toward longer-term supplementation and functional outcomes. A THC-free CBD distillate (4 mg/kg BW/day) was absorbed and generally well tolerated in randomized, blinded, placebo-controlled studies up to 26 weeks, with monitoring suggesting no clinically relevant alterations in hematological or serum biochemical parameters [47]. Evidence for clinical benefit is emerging in disease models. In client-owned cats with osteoarthritis, CBD/CBDA (4 mg/kg BW/day) improved validated pain scores versus placebo in a crossover design without altering biochemical parameters [48]. But the high drop-out rate due to refusal to eat the paste and occasional vomiting is an important limitation for real-world use.

Cannabinoid studies in dogs and cats provide useful guidance on oral dosing safety and clear species differences in exposure, while evidence for clinical benefits is currently strongest for feline osteoarthritis but limited by product acceptance and study drop-outs. More consistent product characterization and practical dosing strategies are needed, especially in cats.

### 2.4. Microalgae/Seaweed

Microalgae and seaweed have gained increasing attention as functional ingredients in pet food due to their abundance in omega-3 fatty acids, proteins, prebiotics, micronutrients, and bioactive compounds. In practice, they are delivered mainly by dietary inclusion or coating on kibble, and studies have focused on three application goals: (i) gut-related endpoints (microbiota/IgA/digestibility), (ii) acceptance and nutrient utilization, and (iii) omega-3 (DHA) enrichment and related metabolic outcomes [49,50,51]. Table 4 summarizes microalgae/seaweed ingredients tested in dogs. Intact seaweeds (*Ascophyllum nodosum*, *Undaria pinnatifida*, *Saccharina japonica*, and *Palmaria palmata*) coated on dog kibble at 15 g/kg of as-is diet for 28 days were well tolerated in healthy adult dogs but had no effect on fecal microbiota, IgA levels, or nutritional digestibility [52]. In contrast, spray-dried microalgae powders (*Chlorella vulgaris*, *Nannochloropsis oceanica*, and *Tetradecimals obliquus*) added at 0.5–1.5% did not affect dietary chemical composition, food intake, fecal output, or apparent total tract digestibility of nutrients and energy in adult Beagles [49]. An increase in protein digestibility was observed only with *C. vulgaris*, but some formulations were less preferred, making palatability and incorporation strategy important practical considerations [49]. For omega-3 enrichment, *Schizochytrium* sp. algal powder or algal oil at 1% for 28 days increased serum DHA levels and antioxidant capacity in adult Beagles while reducing cholesterol and improving coat quality without any adverse liver effects. These results support *Schizochytrium* sp. as a safe and sustainable n-3 PUFA source alternative to fish oil, although evidence remains short-term and breed-limited [53]. For algae/seaweed, inclusion levels should consider processing and palatability, while cat-specific feeding trials remain a key evidence gap.

### 2.5. Others

In companion animal nutrition, single bioactive compounds are increasingly being replaced by integrated formulations that combine plant-derived extracts with complementary functional components to achieve synergistic, multi-target health effects (as shown in Table 4). Fermented glasswort and Ganghwa mugwort plants coated at 1% (*v*/*w*) onto kibble showed higher antioxidant activity in the final diet and acceptable preference in adult Beagles with increased beneficial fecal microorganisms [6]. However, the evidence is mainly based on antioxidant measures in final diet and short preference testing rather than confirmed systemic antioxidant effects in dogs. Dry extruded diets for adult dogs containing 1.5 or 3.0 kg/ton of a yeast cell wall and oregano essential oil blend have potential functional additives for improving intestinal functionality by modulating beneficial fecal genera, leading to greater bacterial diversity [54]. But the higher inclusion reduced diet palatability and dry matter digestibility. In overweight adult dogs, a hepatoprotective supplement containing *Silybum marianum* extract combined with prebiotics, probiotics, omega-3 fatty acids, vitamins, and minerals decreased the overall health condition of the gut after 7 days, which recovered from day 14 onwards, but improved biochemical markers associated with liver function and metabolic status [55]. For mobility support, a double-blind, randomized crossover trial evaluated a supplement containing a green-lipped mussel, curcumin, and blackcurrant leaf extract in client-owned dogs and cats with mild to moderate osteoarthritis. Dogs showed full Canine OsteoArthritis Staging Tool [COAST] score improvement vs. baseline but not vs. placebo, while cats showed improved grooming and playfulness. However, there were no differences observed in Helsinki Chronic Pain Index (HCPI) scores or force plate analysis in dogs, indicating only partial effects [14].

## 3. Functional Roles and Health Impacts

### 3.1. Antioxidant Properties

Evidence from feeding and supplementation studies indicates that plant-derived ingredients can influence oxidative balance in dogs and cats through three main layers: (i) enzymatic antioxidant defense, (ii) redox-related gene expression and liver-associated biomarkers, and (iii) microbial or dietary metabolite production that yields antioxidant active compounds (Table 5). Dietary fortification with rosemary and basil leaf powder increased antioxidant biomarkers (glutathione, superoxide dismutase (SOD), and catalase), reduced oxidative damage markers (malondialdehyde (MDA) and lactate dehydrogenase (LDH)) and enhanced blood glucose regulation in Rottweiler dogs [37]. Curcumin similarly increased antioxidant enzyme activities, non-protein thiols, and total antioxidant capacity while lowering circulating reactive oxygen species (ROS) [33]. However, interpretation should be cautious because part of the trial overlapped with natural infection and antibiotic treatment, and some metabolic markers increased during supplementation. *Vaccinium myrtillus* included in semi-moist canine diets upregulated mitochondrial superoxide dismutase 2 (*SOD_2_*), while *Silybum marianum* reduced plasma alanine transferase (ALT) activity and increased paraoxonase and *SOD_2_* expression, which is consistent with improved hepatic oxidative resilience [35]. However, treatment groups differed in their baseline conditions (healthy vs. arthrosis vs. hepatopathy). These results support a plausible mechanism but do not allow strict comparison of effect sizes across botanicals. Antioxidant support may also be relevant to reproductive resilience. *Lasia spinosa* Thwaites supplementation improving post-thaw motility and viability in male dogs, consistent with reduced oxidative injury during cryopreservation, although direct sperm oxidative damage measures were not reported [30].

Another pathway is the use of diet-formulated natural antioxidants. A blend of essential oils with vitamin E increased glutathione S-transferase and non-protein thiols and reduced circulating ROS, indicating improved oxidative balance without adverse effects on hematological or metabolic health [40]. But the evidence is based mainly on short-term (28-day) biomarker outcomes. Indirect antioxidant modulation can also occur through microbiota-derived metabolites. Marine-derived lipids also provide a complementary antioxidant pathway through omega-3 enrichment and lipid modulation. Algal powder and oil from *Schizochytrium* sp. increased serum antioxidant capacity in Beagles. Supplements reduced oxidative stress indicators, such as trolox equivalent antioxidant capacity and superoxide dismutase activity, but did not affect serum malondialdehyde levels after 28 days of feeding. In comparison to fish oil, 1% algal powder has shown better effects in lowering total cholesterol levels [53]. In summary, antioxidant properties in companion animals reflect multi-level modulation of enzymatic systems, gene expression, and microbiota-derived metabolites, but comparability across studies remains limited by heterogeneous endpoints and frequent reliance on surrogate markers [33,35,37,56].

### 3.2. Anti-Inflammatory and Immunomodulatory Effects

Plant-derived bioactives can influence inflammation and immune regulation through coordinated changes in immunometabolism, cytokine and inflammatory signaling, and gut–immune axis modulation, which are important for maintaining and resolving the host inflammatory response (Table 6). Through dog and cat studies, evidence is most often based on inflammatory gene expression, circulating immune markers, immune cell profiles, and acute-phase proteins rather than clinical endpoints, so findings are best interpreted as mechanistic support. Black ginseng studies show serum metabolite shifts consistent with immunometabolic modulation (e.g., amino acids related to immune/energy pathways and reduced formate), and higher extract exposure increased glycine and β-alanine, proposed as candidate anti-inflammatory biomarkers [26,27]. However, these findings are primarily metabolomics-based and do not directly confirm measured inflammatory cytokines, acute-phase proteins, or clinical outcomes in the same animals. Clear evidence for cytokine-related regulation from studies showed coordinated shifts in inflammatory gene expression along with acute-phase markers. *Curcuma longa* and *Vaccinium myrtillus* reduced Tumor Necrosis Factor alpha (*TNF*), C-X-C motif chemokine ligand 8 (*CXCL8*), nuclear factor kappa B subunit 1 (NFKB1), and prostaglandin-endoperoxide synthase 2 (*PTGS2*) expression and plasma ceruloplasmin, which is consistent with a lower or suppressed inflammatory response profile [35]. Echinacea angustifolia showed a mixed profile; it reduced *TNF* and *NFKB1* expression but increased *CXCL8*, consistent with immune activation in dogs [35]. An oral *Echinacea angustifolia* trial reported enhanced immune responsiveness by increasing leukocyte counts, lymphocytes, phagocytic activity, and IgM concentration, suggesting enhanced immune response [29]. However, larger studies are needed to confirm consistency across breeds and health states, reproducibility, and clinical relevance.

Systemic inflammation is also reflected in acute-phase proteins and liver/metabolic biomarkers, particularly in obesity-related settings. In cats, a quercetin-rich botanical mixture reduced serum amyloid A as an inflammatory marker in both healthy and obese cats. In obese cats, total cholesterol and liver enzymes (AST/ALT) also decreased, which is consistent with reduced inflammatory status and improved lipid- and liver-related markers, although hepatic lipid metabolism was not measured directly [32]. In overweight dogs, a multi-ingredient supplement containing *Silybum marianum* extract, prebiotics, probiotics, n-3 polyunsaturated fatty acids, vitamins, and minerals was associated with reduced C-reactive protein (CRP) and improved metabolic/liver-related biomarkers (decreases in ALP, glucose, and direct bilirubin), although marked individual variability and complex microbiota responses require further investigation [55].

Dietary bioactive compounds modulate immune cell activity and inflammatory cytokine balance, resulting in enhanced immunoglobulin production and anti-inflammatory response with improved systemic immune regulation in companion animals [55,57]. In kittens, enzymolysis seaweed powder reduced pro-inflammatory cytokines (interleukin-1β (IL-1β), IL-6, and tumor necrosis factor-α (TNF-α) levels) while increasing serum IL-10 levels along with barrier-related improvements, supporting anti-inflammatory effects in a developing gut [58].

**Table 6:** Anti-inflammatory and immunomodulatory mechanistic outcomes of plant-derived ingredients in dogs and cats.

Animal Species and Breed	Active Compounds	Key Mechanistic Outcome	Reference
Male and female dogs (2–3-year-old Beagles)	Black ginseng extract (*Panax ginseng*)	Significant serum metabolite discrimination pre- vs. post-supplementation (multivariate profile shift). Key discriminant metabolites included formate, glutamine, histidine, and branched-chain amino acids (valine, leucine, and isoleucine).	[27]
Male and female dogs (2–3-year-old Beagles)	Black ginseng extract (*Panax ginseng*)	Black ginseng extract altered amino acid profiles (alanine, leucine, isoleucine, and valine) versus control. High-dose group showed increased glycine and β-alanine versus control and medium-dose groups (candidate anti-inflammatory biomarkers).	[26]
Multiple dog breeds across four locations, including crossbreeds, German Shepherds, English Setters, and others (> 2-year-old adult dogs)	Nutraceutical extracts: *Vaccinium myrtillus*, *Curcuma longa*, *Echinacea angustifolia*	*Echinacea angustifolia* reduced *TNF* and *NFKB1* and increased zinc, indicating immune modulation. *Vaccinium myrtillus* and *Curcuma longa* reduced ceruloplasmin (acute-phase/oxidative stress-linked marker).	[35]
Male dogs (mixed-breed dogs)	Hydro-alcoholic *Echinacea* extract	Increased packed cell volume, hemoglobin, red blood cells, and white blood cells. Increased lymphocytes and neutrophils at specific time points. Higher percentage of phagocytosis and increased IgM concentration.	[29]
Male and female cats (2–3 years old, mixed breed)	*Rhus verniciflua*, *Ulmus hollandica*, *Polygonatum sibiricum*, *Lycium chinense*, *Ganoderma japonicum*, *Parnax ginseng*	Decreased serum amyloid A (SAA) in healthy and obese cats. Decreased NEFA in healthy cats. Decreased total cholesterol and AST/ALT in obese cats.	[32]
Male and female dogs (8–13-year-old adults; various breeds, including American Staffordshire terrier, Dachshund, English Bulldog, Shar Pei, Staffordshire bull terrier, Beagle, Bullterrier, Dalmatian, German shepherd and Basset hound)	*Silybum marianum* extracts, prebiotics, probiotics, n-3 polyunsaturated fatty acids, minerals, and vitamins	ALP, glucose, direct bilirubin, and CRP decreased (from day 21 to day 35). Fecal microbiota alpha-diversity decreased on day 7. Total short-chain fatty acids and lactic acid concentrations were lower on day 7 compared to all other time points.	[55]
Male and female cats (6-month-old kittens, Ragdoll breed)	Enzymolysis seaweed powder (prebiotic) and comparative ingredient:(*Saccharomyces boulardii* as probiotic)	Decreased inflammatory factors: interleukin-1β (IL-1β), IL-6, and tumor necrosis factor-α (TNF-α). Enriched beneficial taxa (e.g., *Bacteroidetes*, *Lachnospiraceae*, *Prevotellaceae*, and *Faecalibacterium*).	[58]

Note: In the “Key Mechanistic Outcome” column, only outcomes reported as statistically significant versus the appropriate control (*p* < 0.05) are listed.

### 3.3. Gut Health and Microbiota Modulation

Plant-derived functional ingredients can modulate the gut microbiota of dogs and cats by enriching beneficial microbes and altering microbial functions (fermentation products, bile acids, and enzyme activities) that interact with host barrier and immune signaling (Table 7). Orally administered grape seed proanthocyanidin improved intestinal inflammation in dogs with mild IBD and increased beneficial taxa and reduced intestinal permeability consistent with anti-inflammatory and bile-acid metabolism [21]. Fecal microbiota transplantation reproduced benefits, confirming that gut microbial composition alterations contributed to improved canine intestinal health [21]. Mandarin orange peel intake shifted selected taxa (reduced *Fusobacteriaceae* and increased *Eggerthellaceae*) and was consistent with reductions in anxiety-related behaviors and improved activity levels in senior dogs [23]. The behavioral findings are preliminary (*n* = 4), and the study design could not determine whether the behavioral changes were driven by microbiota modulation or by other uncontrolled factors. Under stress conditions, gallnut tannic acid reduced stress-related diarrhea and promoted beneficial genera (*Allobaculum*, *Dubosiella, Coriobacteriaceae*_UCG-002, and *Faecalibaculum*) toward a more saccharolytic profile (higher butyrate) in dogs [5]. But the evidence is limited by the small sample size and short duration, and the reported bacteria–metabolite associations need confirmation.

In high-fat diet-fed dogs, green tea polyphenols were consistent with microbiota shifts (decreased *Bacteroidetes* and *Fusobacterium* and increased Firmicutes) and reduced intestinal inflammatory signaling (including inhibition of TLR4 pathway and lower pro-inflammatory cytokines), supporting an association between microbiota changes and immune signaling than taxonomy alone [13]. In overweight cats, unripe avocado extract changed fecal microbiota structure and fecal metabolites (including tryptophan-related compounds), which is consistent with altered microbial metabolic activity [12].

For odor control, rosemary extract (<100 Da fraction) reduced ammonia and hydrogen sulfide emissions in cats and was associated with increased *Bifidobacterium*, reduced sulfate-reducing bacteria, and lower urease/uricase activity, supporting a direct microbiota–metabolism pathway for odor reduction [38]. However, the specific bioactive molecules responsible for these effects remain unclear. In vitro fermentation, yucca extract and chestnut tannins support the same “metabolic pathway” framing by showing reductions in selected volatile and potentially harmful metabolites, but translation to in vivo outcomes still requires feeding confirmation [39]. Additional evidence from non-companion animal models also suggests that dietary supplementation with probiotics and prebiotics (including the probiotic *Saccharomyces cerevisiae* and a *Pediococcus acidilactici* and yeast cell wall prebiotic extract) can improve intestinal histomorphology and shift microbial community structure [59]. However, species differences limit direct translation to dogs and cats.

Marine-derived ingredients show variable responses. Intact seaweeds had no influence on fecal microbial parameters, metabolites, or intestinal IgA, and had no effect on the relatively high digestibility noted in healthy adult [52]. In contrast, enzymolysis seaweed powder can promote gut health in kittens by increasing the abundance of beneficial gut microbiota, including *Bacteroidetes*, *Lachnospiraceae*, *Prevotellaceae*, and *Faecalibacterium*. It also helps enhance gut barrier function by reducing plasma levels of D-lactate, lipopolysaccharide, diamine oxidase, and intestinal fatty acid-binding protein [58]. Although microalgae supplementation had no effect on adult dog food intake, fecal output, nutrient and energy digestibility, or metabolizable energy, it did reduce the number of defecations and promote beneficial genera such as *Turicibacter* and *Peptococcus*, which are associated with gut health and immunity activation [49]. Finally, synergistic formulations and processing strategies can shift both microbial composition and fermentation outputs. Oregano essential oil combined with yeast cell walls increased fecal bacterial alpha-diversity and decreased histamine, ammonia, and phenols in the feces, but it may reduce palatability or digestibility at higher inclusion levels [54]. In contrast, fermented botanicals showed higher diet preference and increased beneficial gut bacteria composition [6]. These results support that gut health modulation in companion animals is achieved through combined changes in microbial composition, metabolic pathways, fermentation profiles, and barrier permeability.

**Table 7:** Gut health mechanisms: microbiota, fermentation metabolites, and barrier-related markers reported in dogs and cats.

Animal Species and Breed	Active Compounds	Key Mechanistic Outcome	Reference
Male and female dogs (4.32 ± 1.71-year-old adult Labrador Retrievers)	Grape seed proanthocyanidin (GSP)	Improved inflammatory indexes and reduced intestinal permeability. Increased anti-inflammatory-associated bacterial taxa (e.g., *Ruminococcaceae* and *Faecalibacterium*). Increased beneficial bile acids, including chenodeoxycholic acid and lithocholic acid. Fecal microbiota transplant reproduced benefits, confirming microbiota-driven effects.	[21]
Dogs (Beagle; additional demonstration in Shiba Inu and Miniature Dachshund)	Mandarin orange peel extract contains flavonoids (particularly hesperidin and nobiletin)	Gut microbiota changes: increased *Fusobacteriaceae*, decreased *Eggerthellaceae*.	[23]
Male dogs (breed not specified)	Green tea polyphenol (GTP) extracts included catechin (C), EGC, gallic acid, tetrahydrofuran (THF), EGCG, (−)-gallocatechin gallate, ECG, and tea caffeine	Diet-associated gut microbiota shifts (decreased *Bacteroidetes* and *Fusobacteria*; increased *Firmicutes*). Reduced pro-inflammatory cytokines (TNF-α, IL-6, IL-1β) and inhibited TLR4 signaling.	[13]
Dogs (Beagle)	Hydrolysable tannin (tannic acid powder from gallnut)	Reduced stress-related diarrhea. Increased butyrate and enriched beneficial taxa (*Allobaculum*, *Dubosiella*, *Coriobacteriaceae*_UCG-002, *Faecalibaculum*). Reduced opportunistic/pathogenic taxa (*Escherichia-Shigella*, *Streptococcus*).	[5]
Male naturally overweight/obese purpose-bred domestic shorthair cats	D-mannoheptulose-enriched avocado extract	Avocado extract decreased Firmicutes vs. baseline. Avocado extract altered gut microbiota composition, increasing beneficial taxa. Fecal metabolites changed (tryptophan, indole-3-acetate, nicotianamine, glycyl-proline).	[12]
Adult British shorthair cats	Rosemary extract	Reduced ammonia and hydrogen sulfide emissions. Increased *Bifidobacterium* and reduced sulfate-reducing bacteria. Reduced urease/uricase activities (lower nitrogen-related odor metabolites).	[38]
Dogs: mixed-breed, healthy adults Cats: domestic European shorthair, healthy adults	Plant saponins (*Yucca schidigera* extract) and/or hydrolysable tannins (chestnut wood tannins)	In canine fecal cultures, chestnut tannins reduced ammonia and the combined treatment reduced cadaverine. In canine fecal cultures, chestnut tannins reduced sulfur-related volatile compounds during fermentation. In feline fecal cultures, chestnut tannins reduced indole, consistent with lower proteolytic fermentation by-products. Yucca extract increased enterococci counts in canine cultures, while chestnut tannins reduced *Escherichia coli* counts.	[39]
1 and 6 years of age, five small to medium-sized cross-breed dogs: three Border Collies, one Australian Shepherd and one Labrador Retriever	Brown algae (*Ascophyllum nodosum*, *Undaria pinnatifida*, and *Saccharina japonica*) and red alga (*Palmaria palmata*)	Increased calcium digestibility, driven by a significant improvement with *Ascophyllum nodosum* (61.8% vs. 40.1%).	[52]
Male and female cats (6-month-old kittens, Ragdoll breed)	Enzymolysis seaweed powder (prebiotic) and comparative ingredient: (*Saccharomyces boulardii* as probiotic)	Increased beneficial gut taxa, including *Bacteroidetes*, *Lachnospiraceae*, *Prevotellaceae*, and *Faecalibacterium*, relative to the control and basal diet mixed *S. boulardii* groups. Improved gut barrier function-related markers (decreased d-lactate (D-LA), lipopolysaccharide (LPS), diamine oxidase (DAO), and intestinal fatty acid-binding protein (iFABP) levels)).	[58]
Male and female dogs (2.2 ± 0.03-year-old adults, beagle dogs)	Three microalgae species (*Chlorella vulgaris*, *Nannochloropsis oceanica*, and *Tetradesmus obliquus*)	Protein digestibility increased with *Chlorella vulgaris* supplementation. Palatability was reduced at 1.5% inclusion for *C. vulgaris* and *Nannochloropsis oceanica* but not for *Tetradesmus obliquus*. Microalgae supplementation beneficially modulated fecal microbiota, increasing *Turicibacter* and *Peptococcus*, genera associated with gut health and immune function.	[49]
Male and female dogs (4.5-year-old adults, Beagle, Whippet, and mixed breed)	Yeast cell wall and oregano essential oil (YCO)	Inclusion of yeast cell wall and oregano essential oil reduced intake ratio and reduced dry matter digestibility at the 3.0 kg/ton level. Dogs fed the 3.0 kg/ton YCO diet showed lower fecal histamine, phenol, and ammonia, with higher putrescine and cadaverine concentrations. Both YCO levels increased fecal bacterial diversity and increased beneficial taxa such as *Blautia* and *Faecalibacterium*, while reducing *Streptococcus*.	[54]
Dogs (aged 5–10 years, Beagles)	Fermented medicinal plants (polyphenol-rich botanicals) including turmeric (*Curcuma longa*), glasswort (*Salicornia herbacea*), ganghwa mugwort (*Artemisia princeps*) and mixed blend (turmeric + glasswort + mugwort)	Dogs showed significantly higher food preference for diets containing fermented glasswort and fermented Ganghwa mugwort compared with the control. Fecal microbiota showed increased beneficial bacterial counts in dogs fed fermented plant-supplemented diets compared with the control.	[6]

Note: In the “Key Mechanistic Outcome” column, only outcomes reported as statistically significant versus the appropriate control (*p* < 0.05) are listed.

### 3.4. Cognitive and Neurological Benefits

Plant-derived extracts and bioactive compounds may support cognitive or neurological function through two mechanistic themes: neurodegeneration-related pathways (protein aggregation and cognitive scoring) and neuroactive signaling that alters stress responsiveness or behavior (Table 8). Across the available dog and cat studies, most evidence is based on behavioral questionnaires or short-term observational scoring and selected biomarkers rather than direct brain measurements, so findings are best interpreted as mechanistic support. In elderly dogs, dietary cyanidin-3-O-glucoside was associated with improved cognitive dysfunction scores and lower serum amyloid-β oligomers, without adverse clinical effects [25]. This supports a neuroprotection hypothesis, although inflammatory and antioxidant markers were not significantly changed and brain-level mechanisms were not directly measured. Cannabinoid studies mainly inform neuromodulation and tolerability rather than confirmed cognitive enhancement. In Beagle-cross dogs, a 1:20 THC:CBD extract caused dose-dependent neurological responses, with high doses inducing hyperesthesia and ataxia, while low–medium doses produced mild or no behavioral alterations [44]. Conversely, 50:50 CBD/CBDA supplementation did not cause behavioral or neurological alterations in dogs, while cats displayed mild transient responses to oil administration. These results highlight species differences in cannabinoid absorption and tolerance without evidence of central nervous toxicity [16]. In cats, single-dose 1:20 THC:CBD cannabis extract resulted in rapid systemic absorption and linear pharmacokinetics in cats, without observable adverse neurological signs. These findings provide pharmacokinetic and safety data supporting the relevance of cannabinoid-based extracts for future investigations targeting feline neurological and cognitive applications [45]. Oral CBD also produced mild sedative effects in healthy cats, as evidenced by increased sedation scores and reduced resistance to handling at 2–8 h post-dosing, while nociceptive thresholds and vital signs remained unchanged. These findings indicate short-term calming effects without clinically relevant central nervous system depression [46]. Thus, evidence is strongest for biomarker-linked cognitive signals in aged dogs [25] and for dose-dependent neuromodulation and safety/tolerability in cannabinoids [16,44,45,46], but cognitive benefits remain limited, and future studies should use standardized cognitive endpoints with consistent dosing and longer follow-up.

### 3.5. Skin, Coat, or Allergy Support

Plant-derived ingredients may support dermatological health in companion animals through three main pathways: (i) epidermal lipid barrier support, (ii) inflammatory itch–lesion modulation in allergic disease, and (iii) reduction of external triggers such as flea formation (Table 9). For barrier lipid support and coat quality, flaxseed and sunflower seeds increased circulating 18-carbon polyunsaturated fatty acids, which is consistent with enhanced epidermal lipid composition. In dogs, this shift in skin lipid profiles was associated with temporary improvements in coat quality and skin condition [34]. DHA-rich algal ingredients also improved coat morphology traits, suggesting that omega-3 enrichment can influence coat structure and shine, but the trials were short and mainly based on coat scoring rather than barrier markers [53]. In dogs with atopic dermatitis, a diet containing *Siraitia grosvenorii* residual extract reduced transepidermal water loss (TEWL) and improved pruritus and lesion scores, supporting barrier-related and anti-pruritic effects [36]. In cats, reducing flea burden can indirectly lessen pruritus by lowering bite-associated inflammation. In a long-term feeding trial, the Bioticks^®^ botanical blend was well tolerated and progressively reduced flea counts in naturally infested cats, which may support improved skin comfort over time [18].

### 3.6. Metabolic and Cardiovascular Health

Plant-derived ingredients may influence metabolic and cardiovascular health through lipid/adipokine regulation, glucose handling, vascular resilience during stress, and urinary tract health (Table 10). Pea-based diets (with or without *Candida utilis* fermentation) lowered plasma triglycerides, cholesterol, and leptin in both dogs and cats compared with corn-based control diets, which is consistent with improved metabolic status [15]. *Melissa officinalis* supplementation improved stress-related behavior and was linked to altered metabolomic pathways related to lipids and bile acids, suggesting systemic metabolic adaptation, although clinical cardiometabolic outcomes were not directly assessed [28]. In an acute hemorrhage challenge, resveratrol improved blood pressure tolerance without increasing bleeding risk, but did not improve kidney injury markers, indicating a hemodynamic effect rather than renal protection [20]. In dogs with urolithiasis, *Elymus repens* supplementation reduced urinary crystalluria and improved urinary environment markers with stable safety serum renal and electrolyte parameters, supporting short-term urinary metabolic regulation [31]. Finally, long-term THC-free CBD at 4 mg/kg/day was generally well tolerated in healthy cats for 26 weeks, with routine monitoring suggesting no clinically meaningful changes, while liver enzyme monitoring remains a reasonable precaution in cats with hepatobiliary risk [47]. Current evidence supports plant-derived ingredients as tools to modulate lipid/adipokine markers and selected condition-specific outcomes (hemodynamic resilience and urinary indices), but comparability across studies remains limited by differences in endpoints and study models.

## 4. Conclusions

This review synthesizes evidence on plant-derived functional ingredients used in dog and cat nutrition, with an emphasis on how ingredients are applied (dietary inclusion or oral supplementation), effective dosing ranges, and outcomes from controlled feeding trials. Polyphenols and flavonoids are frequently associated with reduced oxidative and inflammatory biomarkers, microbiome modulation, and changes in fermentation-related metabolites. Microalgae and seaweed provide omega-3 fatty acids, particularly DHA, which contribute to lipid metabolism, cardiovascular wellness, and improved skin condition. Combinations of botanical extracts with complementary bioactives show immunomodulatory and metabolic signals that depend on the diet matrix and baseline health status. Cannabinoid studies primarily define pharmacokinetics and tolerability. Dogs exhibit dose-dependent responses influenced by plasma cannabinoid levels and may show neurological signs at high doses. In contrast, cats demonstrate lower oral absorption but generally tolerate long-term supplementation and show clear pain-relieving effects in osteoarthritis. Conclusions are constrained by the limited number of controlled feeding trials in dogs and cats, heterogeneity in experimental design, differences in ingredient form and characterization, variation in dose and duration, and small sample sizes. Larger sample sizes, longer-term studies, and more standardized feeding trials with clinically relevant outcomes are needed to support more precise and evidence-based formulation of functional pet foods.

## Figures and Tables

**Table 3:** Cannabinoid-based ingredients in dogs and cats: oral delivery, exposure/tolerability, and reported functional outcomes.

Functional Ingredient	Mode of Incorporation/Usage	Supplementary Form	Functional Ingredient Dose	Experimental Diet	Targeted Function	Reference
Cannabidiol and cannabidiolic acid from hemp	Administered orally	Canine whole-plant CBD-infused soft chew and oral feline CBD-infused fish oil	Dogs received 2 mg/kg cannabidiol plus cannabidiolic acid twice per day for 84 days Cats received 2 mg/kg cannabidiol plus cannabidiolic acid twice per day for 84 days (total 4 mg per kg per 24 h)	Dogs; cats; cannabidiol (CBD); cannabidiolic acid (CBDA); soft chews; CBD-infused fish oil; 2 mg/kg twice daily; 4 mg/kg/day total; 84-day administration	Long-term tolerability (healthy dogs/cats) Administration acceptance differences	[16]
Cannabis herbal extract (CHE) containing 1:20 ratio of 19-tetrahydrocannabinol (THC):cannabidiol (CBD)	Administered orally	Liquid	Low: 2 mg CBD + 0.1 mg THC/kg bw Medium: 5 mg CBD + 0.25 mg THC/kg bw High: 10 mg CBD + 0.5 mg THC/kg of bw	Beagle-cross dogs (*n* = 13); Phase 1 randomization (medium dose: 5 mg CBD + 0.25 mg THC/kg; high dose: 10 mg CBD + 0.5 mg THC/kg; *n* = 6/group); Phase 2, low dose (2 mg CBD + 0.1 mg THC/kg; *n* = 6); overnight fasting; post-dose sampling; individual/group housing; feeding resumed after sampling	Dose-dependent neurological tolerability profile Adverse neurological signs at high dose (hyperesthesia/ataxia)	[44]
Cannabis herbal extract (CHE) containing 1:20 THC:CBD	Administered orally	Cannabis herbal extract (CHE) with nominal concentrations of 20 mg CBD and 1 mg THC per mL in olive oil base	Low (2 mg CBD + 0.1 mg THC/kg bw) High (5 mg CBD + 0.25 mg THC/kg bw)	12 healthy adult cats; stratified by weight and sex; randomized (low-dose *n* = 6, high-dose *n* = 6); 12 h fasting; single oral dose; low dose (2 mg CBD + 0.1 mg THC/kg); high dose (5 mg CBD + 0.25 mg THC/kg); feeding resumed 2 h post-dose; blood sampling up to 48 h; pharmacokinetics	Approximately linear dose–exposure under fasting No observed neurological adverse signs (single dose)	[45]
Cannabidiol	Administered orally	CBD oil in a capsule (CBD:THC ≥ 20:1)	Oral dose: 8 mg/kg of CBD Single-dose administration	9 cats; single dose; 8 mg CBD/kg; capsule administration; 2 h post-meal; no placebo; no control group	Mild sedation Improved compliance during handling No change in nociceptive threshold	[46]
Cannabidiol	Administered orally	THC-free CBD in placebo oil incorporated into a commercial pate (Purina^®^ Gourmet Gold) food	4 mg/kg bw per day	Placebo control (sunflower oil + flavoring, no cannabidiol); complete and balanced commercial diet; ideal body weight/BCS maintenance; 20 healthy adult cats; 26-week study; daily 8 g bolus; commercial pâté (Purina Gourmet Gold); pre-morning feeding	Long-term tolerability (stable clinical chemistry/hematology) Measurable systemic exposure with repeated dosing ALT monitoring signal (transient increase in short study)	[47]
Cannabidiol (CBD) and cannabidiolic Acid (CBDA)	Administered orally	Cannabidiol/cannabidiol acid-rich hemp paste	4 mg/kg BW per day (combined CBD + CBDA) Divided into two equal doses daily for 6 weeks per phase	Randomized; double-blind; placebo-controlled; crossover design; CBD/CBDA paste; 6-week treatment; 2-week washout; reverse sequence; self-control design (within-subject)	Pain modulation (reduced osteoarthritis pain scores) Support of joint comfort Improved mobility/activity-related behavior	[48]

Note: In the “Targeted Function” column, only outcomes reported as statistically significant versus the appropriate control (*p* < 0.05) are listed.

**Table 4:** Microalgae/seaweed and multi-component plant-derived blends in pet foods: delivery format and reported functional outcomes.

Functional Ingredient	Mode of Incorporation/Usage	Supplementary Form	Functional Ingredient Dose	Experimental Diet	Targeted Function	Reference
**Microalgae/Seaweed**						
Brown algae (*Ascophyllum nodosum*, *Undaria pinnatifida*, and *Saccharina japonica*) and red alga (*Palmaria palmata*)	Seaweed powders were spread over kibble together with a small amount of water to ensure their adhesion and total consumption	Dry dog food (kibble)	Seaweeds were added to food at a daily dose of 15 g/kg of as-is diet	10 healthy adult dogs; 5 × 5 replicated Latin square; commercial extruded control diet (CD); CD + *Ascophyllum nodosum*; *Undaria pinnatifida*; *Saccharina japonica*; *Palmaria palmata*; 30-day adaptation; 28-day feeding periods; 7-day washout; fecal sampling (days 21, 28); pooled samples (days 24–28); chemical and microbiological analyses; ATTD evaluation	No significant effects on fecal microbiota, fecal IgA, or digestibility at 15 g/kg	[52]
Three microalgae species (*Chlorella vulgaris*, *Nannochloropsis oceanica*, and *Tetradesmus obliquus*)	Spray-dried powder was manually added to the reference diet immediately before feeding, thus not being incorporated into the reference diet kibble	Dry extruded dog food	Palatability tests: 1.5% added to reference diet Digestibility tests: 0.5, 1.0, and 1.5% added to reference diet	Palatability test; 12 dogs; three two-bowl trials; reference diet vs. 1.5% microalgae inclusion; digestibility trial; replicated Latin square design; 6 dogs per microalga; 3 periods; inclusion levels (0.5%, 1.0%, 1.5%)	Improved protein digestibility (*C. vulgaris*) Selective microbiota enrichment (*Turicibacter* and *Peptococcus*) Reduced diet preference at high inclusion (1.5%)	[49]
Microalgal from *Schizochytrium* sp.	Algal powder or algal oil was incorporated into dry food	Dry extruded dog food	1% algal powder or 1% algal oil	24 healthy adult Beagles; randomized design; four dietary groups; 28-day feeding trial; control (no omega-3); 1% supplementation (fish oil, algal powder, algal oil); nutritionally comparable diets; equal tocopherol antioxidant control	Increased serum DHA Improved antioxidant capacity Improved coat quality Reduced total cholesterol (algal powder)	[53]
**Others**						
Fermented medicinal plants (polyphenol-rich botanicals): turmeric (*Curcuma longa*), glasswort (*Salicornia herbacea*), ganghwa mugwort (*Artemisia princeps*) or mixed blend (turmeric + glasswort + mugwort)	Fermented plants were sprayed at 1% (*v*/*w*) onto extruded dog food before final oil coating	Dry extruded dog food	Fermentation substrate concentrations: turmeric (5%, *w*/*v*), glasswort (2.5%, w/v), Ganghwa mugwort (2.5%, *w*/*v*), their mixture (5%, *w*/*v*) and autochthonous *E. faecium* (1%, *v*/*v*)	Commercial dry control diet (200–300 g/day pre-trial); two-pan test (500 g control + 500 g test diet); no additional foods; 30 min feeding period; intake measured (spillage + leftovers weighed); daily bowl position alternation (side-bias control); water ad libitum; 30 min daily outdoor access	Enhanced antioxidant capacity of dog food Improved food palatability Increased beneficial fecal microorganisms	[6]
Yeast cell wall and oregano essential oil	Added by coating on the kibble surface after extrusion, together with oil and liquid palatant	Dry extruded dog food	Diet containing 1.5 kg/ton of yeast cell wall and oregano essential oil (1.5YCO) and a diet containing 3.0 kg/ton of yeast cell wall and oregano essential oil	18 healthy adult dogs; individual housing; randomized repeated-measures (20 days); control diet; 1.5 kg/ton additive; 3.0 kg/ton additive; yeast cell wall + oregano essential oil; ≥25 g/kg MOS; ≥5.83 g/kg β-glucans; post-extrusion coating; adult maintenance diet; twice-daily feeding; ad libitum water	Improved fecal fermentation profile (lower histamine, phenol and ammonia) Enriched beneficial genera (*Blautia* and *Faecalibacterium*) Reduce fecal odor Reduced intake ratio and dry matter digestibility (high inclusion)	[54]
*Silybum marianum* extracts, prebiotics, probiotics, n-3 polyunsaturated fatty acids, minerals, and vitamins	Administered orally	Tablets	Daily dose: 1 tablet per 15 kg body weight Tablet weight: 2 g Supplement duration: 35 days	42-day monitoring; commercial maintenance diet; 7-day adaptation; supplementation (1 tablet/15 kg BW/day); no control group; within-subject baseline comparison; NRC ME requirement (110 kcal/kg metabolic BW)	Reduced C-reactive protein (CRP) Improved liver/metabolic biomarkers (ALP, glucose, direct bilirubin) Transient microbiome diversity reduction with recovery	[55]
Green-lipped mussel extract (*Perna canaliculus*), curcumin (*Curcuma longa*) and blackcurrant (*Ribes nigrum*) leaf extract	Administered orally	Dietary supplementation with dose based on body weight following manufacturer schedule (i.) <10 kg: 3 mL/day for 25 days (ii.) 10–30 kg: 5 mL/day for 40 day (iii.) 30–45 kg: 7.5 mL/day for 40 days (iv.) >45 kg: 10 mL/day for 40 days	Synopet^®^ Cani-Syn composition (dry-matter basis; product is ~93% water): 571,400 mg GLMax^®^, green-lipped mussel, 142,900 mg Bio-CM100^®^, Bio-Curcumin, 238,571 mg, blackcurrant leaf extract, 242,857 mg vitamin C and 28,571 IU vitamin D3	32 dogs and 16 cats; client-owned; mild–moderate osteoarthritis; double-blind, randomized, placebo-controlled crossover design; 16-week periods in dogs and 10-week periods in cats with a 2-week washout between periods	Improved grooming and playfulness (cats) Improved activity/stair climbing (cats)	[14]

Note: In the “Targeted Function” column, only outcomes reported as statistically significant versus the appropriate control (*p* < 0.05) are listed.

**Table 5:** Antioxidant-related mechanisms and biomarkers reported for plant-derived ingredients in dogs and cats.

Animal Species and Breed	Active Compounds	Key Mechanistic Outcome	Reference
Rottweiler dogs (4 months of age at the start and 6 months at the end)	Rosemary (*Rosmarinus officinalis*) or basil (*Ocimum basilicum*) leaf powder	Basil (0.05%) lowered serum glucose (~31%). Rosemary + basil reduced glucose by 16.25%, and rosemary alone reduced glucose by 14%. Basil reduced amylase activity, suggesting reduced carbohydrate breakdown. Basil increased insulin and decreased cortisol, supporting improved glycemic regulation. All supplemented groups showed increased antioxidant biomarkers (glutathione, SOD, catalase) and reduced MDA and LDH.	[37]
Male dog (4-month-old Beagles)	Curcumin extract powder	Curcumin reduced feed protein/lipid peroxidation and increased total antioxidant capacity. Increased serum glucose, urea, triglycerides, and cholesterol. Higher red blood cell counts at day 35 and day 45. Increased white blood cells (neutrophil-driven) and reduced lymphocytes (anti-inflammatory signal). Increased antioxidant enzymes, non-protein thiols, and serum total antioxidant capacity with lower reactive oxygen species.	[33]
Multiple dog breeds across four locations, including crossbreeds, German Shepherds, English Setters, and others (>2-year-old adult dogs)	Nutraceutical extracts: *Vaccinium myrtillus*, *Curcuma longa*, *Sylibum marianum*	*Silybum marianum* lowered alanine transferase (ALT/GPT) and increased paraoxonase with SOD_2_ upregulation (hepatoprotective antioxidant signal). *Vaccinium myrtillus* upregulated antioxidant-related signals (e.g., *SOD_2_*)/antioxidant-associated markers.	[35]
Male dogs (2–3 years, five Labrador retrievers and one German shepherd)	*Lasia spinosa Thwaites*	Ejaculate volume increased significantly during long-term supplementation. Post-thaw sperm motility increased at 15 min and 4 h. Post-thaw sperm viability improved at 4 h after thawing.	[30]
Adult male beagle dogs	Blend of essential oils including clove essential oil, rosemary essential oil, oregano essential oil and vitamin E (α-tocopherol)	Dogs fed the essential oil blend showed a 21.5% reduction in blood ROS levels at day 28 compared with the BHT diet. Fecal total bacterial count decreased by 54.8% at day 28. Lymphocyte counts decreased over time only in treated dogs, remaining within reference ranges.	[40]
Male and female adult Beagle dogs (2 ± 0.31 years)	Microalga from *Schizochytrium* sp.	Algal powder and algal oil significantly increased serum DHA levels compared with fish oil. Algal powder reduced total cholesterol to 69.4% of control. Antioxidant capacity improved without increasing lipid peroxidation.	[53]

Note: In the “Key Mechanistic Outcome” column, only outcomes reported as statistically significant versus the appropriate control (*p* < 0.05) are listed.

**Table 8:** Cognitive and neurological outcomes and associated biomarkers reported for plant-derived ingredients in dogs and cats.

Animal Species and Breed	Active Compounds	Key Mechanistic Outcome	Reference
Neutered male and female dogs (≥7 years old, elderly; mixed breeds, 6 species; small breeds (3.96 kg) to large species (28.00 kg))	Hydrolyzed honeyberry (rich in anthocyanin cyanidin-3-O-glucoside)	Cyanidin-3-glucoside supplementation significantly reduced serum amyloid beta oligomers. Cognitive dysfunction scores improved in elderly dogs.	[25]
Intact female, neutered female, intact male, and neutered male dogs (22–24 months, Beagle cross dogs)	Cannabis herbal extract (CHE) containing 1:20 ratio of 19-tetrahydrocannabinol (THC):cannabidiol (CBD)	High-dose treatment produced clinically meaningful neurological signs (hyperesthesia/ataxia). Severity of neurological signs correlated with plasma cannabinoid concentrations. CBD/THC exposure increased non-proportionally with dose (non-linear dose–exposure).	[44]
Purpose-bred Beagle dogs (11 months to 5 years of age) Purpose-bred domestic shorthair cats (2–6.3 years of age)	Cannabidiol and cannabidiolic acid from hemp	Dogs showed significantly higher systemic CBD exposure than cats (C_max_/AUC). One cat showed a persistent increase in ALT above the reference range throughout treatment, without associated clinical signs. Cats demonstrated markedly lower CBD exposure (lower C_max_ and AUC), indicating reduced oral absorption or faster elimination compared with dogs.	[16]
Castrated male and spayed female cats (0.75–9 years, domestic shorthair cats)	Cannabis herbal extract (CHE) containing 1:20 THC:CBD	Dose-adjusted THC exposure (C_max_/AUC) was significantly higher than CBD (*p* < 0.01).	[45]
Male and female domestic shorthair cats and Persian cats (3.44 ± 2.35 years)	Cannabidiol	Sedation scores increased significantly at 2, 4, and 8 h post-administration. Handling compliance and temperament scores significantly improved at 2–4 h post-dose.	[46]

Note: In the “Key Mechanistic Outcome” column, only outcomes reported as statistically significant versus the appropriate control (*p* < 0.05) are listed.

**Table 9:** Skin/coat/allergy-related outcomes and barrier-associated markers reported for plant-derived ingredients in dogs and cats.

Animal Species and Breed	Active Compounds	Key Mechanistic Outcome	Reference
Male and female dogs (mixed breed)	Flaxseed Sunflower seed	Dogs fed flaxseed showed increased relative percentage of serum 18:3 (n-3). Dogs fed flaxseed also showed increased relative percentage of 18:2 (n-6) in serum phospholipids. The ratio of polyunsaturated fatty acids to saturated fatty acids increased temporarily in both groups.	[34]
Male and female adult Beagle dogs (2 ± 0.31 years)	Microalgal from *Schizochytrium* sp.	Improved coat length and gloss compared with control.	[53]
Male and female dogs (5.3 years, multiple breeds: Poodle, Pomeranian, Maltese, Bichon Frise, others)	*Siraitia grosvenorii* residual extract	Pruritus scores (PVASs) decreased significantly after the functional diet phase. CADESI-04 lesion scores improved markedly compared with baseline. Transepidermal water loss at affected sites decreased, suggesting enhanced skin barrier function.	[36]
Male and female cats (5 months to 19 years, mixed or pure breeds)	Biological plant-based food supplement Bioticks^®^ (thyme, rosemary, lemon balm, fenugreek, wormwood, and lemongrass extracts)	Flea counts decreased steadily from a mean of 21.7 to 3.3 fleas by month five. Flea counts remained stable in the placebo group. Significant reductions in flea numbers occurred from day 90 onward.	[18]

Note: In the “Key Mechanistic Outcome” column, only outcomes reported as statistically significant versus the appropriate control (*p* < 0.05) are listed.

**Table 10:** Metabolic and cardiovascular outcomes and related biomarkers reported for plant-derived ingredients in dogs and cats.

Animal Species and Breed	Active Compounds	Key Mechanistic Outcome	Reference
Neutered/spayed male and female dogs: 3–4-year-old adults, Beagle. Neutered/spayed male and female cats: 2–5-year-old adults, mixed breed.	*Candida utilis*–fermented pea starch	Pea-based diets lowered plasma triglycerides, cholesterol, and leptin in both dogs and cats compared with the corn control diet, indicating improved metabolic status.	[15]
Male and female dogs (2.7 ± 0.1 years, Beagle)	Hydro-alcoholic *Melissa officinalis* extract	Supplementation significantly reduced stress-related behaviors, including hyperactivity and excessive barking. Plasma 4-hydroxybutyric acid (GHB) decreased in supplemented groups compared with placebo.	[28]
Male dogs (2–7-year-old adult retired racing greyhounds)	Resveratrol (micronized trans-resveratrol powder)	Greater volume of blood loss required to reach target hypotension in resveratrol group versus placebo (*p* = 0.041). Baseline clot strength higher in resveratrol dogs versus placebo (*p* = 0.009).	[20]
Male dogs (6–10 years, different breeds)	*Elymus repens* (couch grass) extracts	*Elymus repens* showed a significant reduction in urinary crystal formation and degree of urolithiasis compared with controls. Significant decreases in serum creatinine, uric acid, and blood urea nitrogen were observed after supplementation. Treated dogs showed improved urine specific gravity and pH toward normal ranges, indicating better urinary environment for stone dissolution.	[31]
Cats (domestic cats housed in social rooms)	Cannabidiol	Postprandial plasma CBD exposure increased after 2 weeks of daily dosing (higher AUC (*p* = 0.002) at day 14 vs. day 0).	[47]

Note: In the “Key Mechanistic Outcome” column, only outcomes reported as statistically significant versus the appropriate control (*p* < 0.05) are listed.

## Data Availability

The data presented in this study is available on request from the corresponding author.
